# A multimodal speech-production dataset with time-aligned articulography, EEG, audio, and vocal-tract anatomy

**DOI:** 10.1038/s41597-026-07704-3

**Published:** 2026-07-01

**Authors:** Daniel Friedrichs, Valeriia Vyshnevetska, Monica Lancheros, Elena Bolt, Volker Dellwo, Steven Moran

**Affiliations:** 1 Zurich Forensic Science Institute, Zurich, Switzerland; 2 Department of Computational Linguistics, University of Zurich, Zurich, Switzerland; 3 Linguistic Research Infrastructure (LiRI), University of Zurich, Zurich, Switzerland; 4 Faculty of Psychology and Educational Sciences, University of Geneva, Geneva, Switzerland; 5 Institute of Biology, University of Neuchâtel, Neuchâtel, Switzerland

## Abstract

We present a multimodal speech-production dataset combining simultaneous electromagnetic articulography (EMA), electroencephalography (EEG), and audio from 29 adult native speakers of German. All participants have external craniofacial anthropometry; an anatomical subset (N = 18) additionally contributed acoustic pharyngometry, rhinometry, and 3D head surface meshes. Speech materials include high-trial diadochokinetic sequences at habitual and maximally fast rates, plus EMA+audio for passage reading and sustained vowels, as well as EMA for palate tracing and non-speech oromotor actions. The corpus contains 8,700 syllable-task trials and approximately 17 h of EMA with matched audio. A microcontroller mirrors each EMA sweep’s start and stop as 1 ms transistor—transistor logic (TTL) pulses on the EEG digital Status channel, enabling sub-millisecond alignment. We distribute raw and minimally processed streams, stable event codes, and machine-readable metadata, plus example Python utilities (and a container) for loading, synchronising, and basic preprocessing. The resource supports studies that exploit articulatory landmarks for EEG alignment, examine pre-movement activity, assess overt-speech EEG artefact handling, and develop anatomy-informed models linking vocal-tract structure to articulatory dynamics and acoustics. Data and code are openly available under a CC-BY 4.0 licence with versioned DOIs.

## Background & Summary

Human speech emerges from tightly coordinated neural activity, articulatory biomechanics and speaker-specific vocal-tract anatomy, which is realised as an acoustic signal. Quantitative models of speech production increasingly require data that link these components at high temporal precision. However, most established corpora span only one or two of these domains. Classic articulatory datasets such as XRMB^1^, MOCHA-TIMIT^2^ and mngu0^3^ pair detailed tongue and lip kinematics with audio, but provide no brain signals. Conversely, neuroimaging resources relevant to speech (e.g., intracranial iBIDS^4^ or EEG sets related to KARA ONE^5^) offer neural time series such as electroencephalography (EEG) alongside audio data, but lack direct imaging of internal articulators. Speaker-specific anatomy is likewise seldom documented alongside behaviour, despite evidence that inter-speaker differences in vocal-tract morphology shape articulation and acoustics within and across speech communities^6–8^. As a result, researchers who wish to study neural-articulatory coupling, plan robust artefact handling for overt-speech EEG, or model how static anatomy constrains kinematics must typically combine heterogeneous sources with imperfect alignment and incomplete metadata.

A central barrier to integrated neural-articulatory datasets is timing. The articulatory-acoustic onset interval (AAOI)—the lag between movement onset and the resulting acoustic change—means that analyses time-locked to sound onsets mix preparatory neural activity with facial muscle artifacts and early auditory responses^9–11^. Electromagnetic articulography (EMA) provides direct access to gesture onsets and thus a more appropriate anchor for neural analyses, but only if EMA and EEG are co-registered at sub-millisecond precision and remain free of mutual interference. Even modest offsets or jitter can obscure brain-behaviour relations and attenuate effects when averaged across trials.

Here, we release a multimodal speech-production dataset designed to meet these requirements. We recorded simultaneous EMA, EEG, and high-fidelity audio from twenty-nine adult native speakers of German. For all participants we additionally provide external craniofacial anthropometry (bilateral mandibular segments, mouth-opening measures, head-size measures); an anatomical subset (*N* = 18) contributes acoustic pharyngometry and rhinometry (area-distance profiles of oral-pharyngeal and nasal cavities) together with 3D head surface meshes. Speech materials include diadochokinetic (DDK) syllable sequences at habitual and maximally fast rates with high trial counts, passage reading (*North Wind and the Sun*), sustained vowels in isolation and in context, as well as palate tracing, and non-speech oromotor actions. EEG was recorded for syllable blocks; EMA+audio were recorded for all speech materials. EMA was recorded for palate tracing and non-speech oromotor actions.

Cross-modal timing is provided by microcontroller-driven transistor—transistor logic (TTL) pulses that mark the start and stop of each EMA sweep on the BioSemi Status channel; optional post-hoc audio cross-correlation refines alignment. Bench oscilloscope tests and empirical checks indicate sub-millisecond residual offsets with negligible jitter and no measurable electromagnetic interference between systems^12^. Sampling rates are EMA 1250 Hz, EEG 2048 Hz, and audio 48 kHz. This design enables analyses time-locked to articulatory events (e.g., tongue-tip elevation for /t/) rather than to later acoustic onsets, exposing tens to hundreds of milliseconds of pre-articulatory neural dynamics that are otherwise difficult to isolate^13^.

The choice of DDK materials for EEG is deliberate. Alternating-motion-rate (AMR) and sequential-motion-rate (SMR) sequences sample core phonetic building blocks—labial, alveolar and velar places; voiced and voiceless conditions—under precisely controlled timing with many repetitions. This facilitates robust averaging and statistical modelling of gesture sequencing, rate effects, and preparation intervals while providing a clean test bed for artefact-mitigation approaches (e.g., independent component analysis (ICA) and regression-based methods) in overt-speech EEG^13^. The additional EMA+audio materials (passage reading and isolated/contextual vowels) extend the articulatory and acoustic coverage beyond the constrained syllable tasks. The non-speech oromotor actions and the palate tracing provide complementary control recordings that broaden the articulatory coverage and support, for example, transfer learning and cross-context validation of models trained on DDK behaviour.

The anatomical component adds a speaker-specific layer often missing from speech corpora. Acoustic pharyngometry provides oral-pharyngeal area-distance curves that relate directly to vocal-tract length and cross-sectional shape, while acoustic rhinometry supplies complementary nasal cavity profiles; both methods are established for characterising upper-airway geometry^14,15^. External anthropometry (mandibular segments; mouth-opening ranges; head circumference and linear head measures) and 3D head meshes further support studies of how external and internal craniofacial dimensions covary with articulatory strategy, kinematic range, or planning time, and they enable anatomy-aware normalisation across speakers^7,8^. Together with palate tracings from EMA, these records allow users to interrogate links between static geometry, articulatory motion and acoustics using a single, consistently annotated resource^6^.

To maximise reuse and reproducibility, the deposit provides both raw and minimally processed files, plus machine-readable metadata and documented event codes. At the participant level, EMA sweep identifiers serve as stable basenames that pair 1:1 with per-sweep audio where available and with EEG markers via TTL codes. At the dataset level, we supply data dictionaries, an event-code map, and a session index that links tasks and blocks to EMA sweep numbers, enabling programmatic cross-modal pairing. Example Python utilities and a containerised environment demonstrate (i) parsing BioSemi Status bits to recover sweep start/stop, (ii) aligning EMA-EEG-audio, (iii) computing articulatory onsets from EMA velocities, and (iv) reading pharyngometry/rhinometry exports and reconstructing area-distance profiles. These examples are intended as minimal, auditable starting points rather than prescriptive pipelines.

Potential uses span multiple communities. In psycholinguistics and cognitive neuroscience, the data allow hypothesis tests about preparatory activity relative to movement onset (e.g., go-cue to articulatory-onset intervals; sequencing differences between AMR and SMR; rate-dependent premotor dynamics). In speech motor control, the simultaneous EMA-EEG enables benchmarking of overt-speech artefact handling and supports methodological evaluation relevant to clinical work (although the present dataset contains typical adult speech only), for example by quantifying gesture-timing measures that are widely used in dysarthria and apraxia research. In speech technology, the tightly aligned streams support multimodal modelling (e.g., EEG- or EMA-conditioned acoustic prediction; anatomy-aware acoustic-to-articulatory mapping), while the high-trial-count syllable tasks provide controlled training and validation sets. Finally, the anatomical measurements invite work on how inter-speaker differences in vocal-tract geometry constrain kinematic strategies and acoustic outcomes^6–8^.

Data are distributed for reuse under the Creative Commons Attribution 4.0 International (CC BY 4.0) license and are accompanied by versioned metadata and usage notes. The following sections detail participants, tasks, hardware, synchronisation, preprocessing and file formats (*Methods*); give an inventory of data records and repository layout (*Data Records*); report quantitative validation of cross-modal timing and anatomy/anthropometry quality (*Technical Validation*); and provide practical guidance with runnable code snippets for typical analysis workflows (*Usage Notes*).

## Methods

### Participants

Twenty-nine healthy, right-handed adults (22 female, 7 male; 19–32 yr; median 23.9 yr; mean 24.5 yr ± 3.3 yr (SD)) took part. All were native speakers of German with normal or corrected-to-normal vision and no self-reported history of speech, hearing, or neurological disorders. Written informed consent was obtained in accordance with the Ethics Committee of the Faculty of Arts and Social Sciences at the University of Zurich (approval #24.12.07), and participants received financial compensation. A subset (*N* = 18; 13 female, 5 male) additionally completed acoustic pharyngometry, rhinometry, and 3D head surface scanning; their ages spanned 19—32 yr (median 23.5 yr; mean 24.5 yr ± 3.6 yr (SD)) (Table 1).

**Table 1:** Participant summary.

	All participants (N = 29)	Anatomy subset (N = 18)
Sex (female : male)	22 : 7	13 : 5
Age range (years)	19–32	19–32
Mean age ± SD	24.5 yr ± 3.3 yr	24.5 yr ± 3.6 yr
Handedness	all right-handed	all right-handed
Tasks recorded	EMA, EEG, audio^a^	EMA, EEG, audio; pharyngometry; rhinometry; head scan

Overview of demographics for the full sample and for the subset who also completed anatomical measurements. **Note**^a^: For syllable (DDK) tasks, *tri-modal* coverage (EMA+EEG+*EMA-synced* per-sweep audio) is complete for **25** participants; **26** participants have *EMA-synced* per-sweep audio; and **all** participants have EEG-system prompt+response WAVs (300 files). Exact per-participant flags are provided in docs/metadata.csv.

All participants provided written informed consent for the open sharing of de-identified research data; participants shown in the setup photographs also consented to publication of these illustrative images. The released data repository contains no direct identifiers or participant photographs; participant IDs are pseudonyms (sub-XXX). Surface head scans are provided as 3D meshes without facial texture and are intended for geometric modelling (e.g., sensor-to-head registration) rather than biometric identification.

### Experimental design and tasks

Recordings took place in a sound-attenuated room (ambient noise < 25 dB(A)). Participants were seated upright and asked to minimise head/body movements apart from those required for articulation. For speech tasks, EMA coils and a 64-channel EEG cap were worn throughout, except the first passage reading (baseline without EMA) and the two subsequent passage readings (EMA + audio only; no EEG).

Core speech materials comprised diadochokinetic (DDK) paradigms at two rates: habitual and maximally fast. Alternating motion rate (AMR) trials required rapid repetition of a single syllable; sequential motion rate (SMR) trials alternated across places of articulation. The inventory sampled three places (labial, alveolar, velar) and two voicing conditions:

- AMR (voiceless): */pipipi/*, */tatata/*, */kukuku/*
- AMR (voiced): */bibibi/*, */dadada/*, */gugugu/*
- SMR: */pitaku/*, */takupi/*, */kutapi/*

The DDK tasks are organised into acquisition blocks that correspond to individual AG501 sweeps (normal_block_1..9 at habitual rate and fast_block_1..6 at maximum rate; see docs/session_index.csv). At the habitual rate, nine sequence types × 20 trials yielded 180 trials (interleaved across blocks). At the maximally fast rate, six sequence types (AMR voiceless + SMR) × 20 trials yielded 120 trials. Additional EMA + audio materials included passage reading (*North Wind and the Sun*: one baseline reading without EMA and two readings with EMA), sustained productions of the long German vowels /i, y, e, ø, ɛ, a, o, u/ with two repetitions per vowel in isolation and two repetitions per vowel in a fixed carrier word frame of the form */l-V-gen/* (with V = target vowel), and palate tracing (two structural palatal surface sweeps). We also recorded non-speech oromotor actions (two maximal tongue protrusions and two swallows). The vowel recordings were designed as supplementary breadth-oriented EMA + audio coverage rather than as a high-repetition vowel corpus. The DDK tasks provide the high-trial-count component. Total session duration including breaks was ~90 min.

**Fig. 1:**
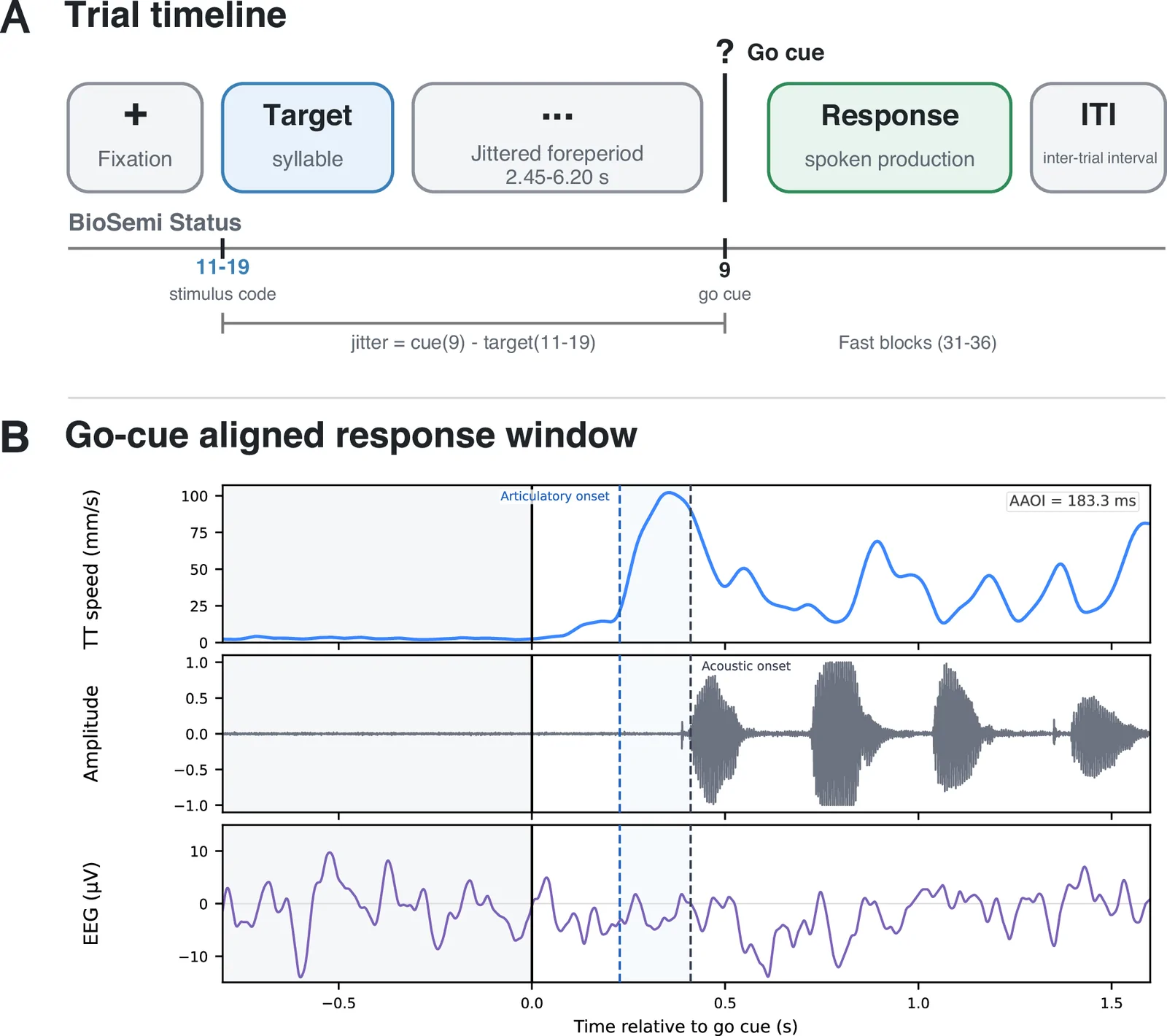
Task timeline and cross-modal alignment (example: sub-125). **(A)** Trial timeline for a cue-based DDK trial. A fixation cross is followed by the target sequence, a jittered foreperiod displayed as an ellipsis (2.45–6.20 s), and a question mark that serves as the explicit go cue, after which the participant produces the spoken response and the trial ends with an inter-trial interval. In the BioSemi Status channel, stimulus onsets are coded as 11–19 and the go cue as 9; fast blocks (31–36) omit the separate go cue. **(B)** Go-cue-aligned response window (*t* = 0 at the go cue) showing tongue-tip (TT) speed, audio amplitude, and EEG for the same trial. The dashed blue line marks articulatory onset, the dashed black line marks acoustic onset, and the shaded interval between them is the articulatory–acoustic onset interval (AAOI). The display highlights the pre-acoustic interval available for alignment to articulatory landmarks.

Stimuli were presented on a monitor. Within each sweep, a fixation cross (“+”), the target sequence, and an ellipsis ("...”) preceded a “?” that served as the explicit go cue in *habitual-rate* blocks. The foreperiod duration was jittered (randomised) to reduce temporal expectancy and ranged from 2.45–6.20s (mean 4.87 s, SD 0.95 s). Event codes followed a stable schema written to the BioSemi Status channel (Table 2; see also docs/event_codes.csv): habitual stimuli use codes 11–19 with a separate go cue (code 9), whereas maximally fast blocks use codes 31–36 (no separate go cue). In habitual-rate trials, the foreperiod jitter is recoverable as the interval between the stimulus code (11–19) and the subsequent go-cue code 9 in the EEG Status channel. Within each normal_block_* or fast_block_* sweep, the prompted trials occur sequentially in a pseudorandom order; the trial identity is therefore recovered from the Status task codes rather than from the block label. Sweep boundaries were marked by 1 ms TTL pulses on bit 6 (start, value 64) and bit 7 (stop, value 128). Figure 1 summarises the single-trial timeline and illustrates the alignment of articulatory, acoustic, and EEG signals for a representative sweep.

**Table 2:** EEG event codes in the BioSemi Status channel and their meanings.

Raw value(s)	Meaning
64 (bit 6); 73 (=64+9)	EMA sweep *start* TTL pulse
128 (bit 7); 137 (=128+9)	EMA sweep *stop* TTL pulse
9	Go cue
11, 12, 13	Habitual AMR stimulus (*/pipipi/*, */tatata/*, */kukuku/*)
14, 15, 16	Habitual SMR stimulus (*/pitaku/*, */takupi/*, */kutapi/*)
17, 18, 19	Habitual AMR voiced stimulus (*/bibibi/*, */dadada/*, */gugugu/*)
31, 32, 33	Maximally-fast AMR stimulus (*/pipipi/*, */tatata/*, */kukuku/*)
34, 35, 36	Maximally-fast SMR stimulus (*/pitaku/*, */takupi/*, */kutapi/*)

Rising edges on bit 6 (=64) mark EMA sweep starts; rising edges on bit 7 (=128) mark sweep stops. Task codes are recovered via *v*_core_ = *v* *&* 63. Because TTL bits (start = 64, stop = 128) can coincide with task codes on the same sample, composite integers appear (e.g., 73 = 64+9, 137 = 128+9). Decoding examples and bit-mask utilities are provided in *Usage Notes*.

### Acquisition

#### Electromagnetic articulography (EMA)

Articulatory kinematics were recorded with a Carstens AG501 electromagnetic articulograph^16^ at 1250 Hz. The AG501 is the laboratory EMA platform used for this corpus and, to our knowledge, the currently available commercial EMA option for speech-production laboratories. Other EMA systems relevant to the field include the NDI Wave speech research system^17^, which has been discontinued but remains in use, and MagTrack^18^, a wearable tongue-motion tracking system under active development. Electromagnetic articulography estimates articulator motion by tracking small sensor coils attached to speech articulators inside low-intensity alternating magnetic fields generated by a transmitter array. The voltages induced in each coil are used to reconstruct its pose at high sampling rates, yielding millimetre-scale trajectories during speech. For each AG501 sensor, the system provides three Cartesian coordinates (*x*, *y*, *z*) and two orientation angles (*ϕ*, *θ*).

Across the session we used 12 AG501 channels: coils on the upper and lower lips, a left lip corner, jaw proxies (upper and lower incisors), and three midline tongue sensors (dorsum, blade, tip), plus three reference coils (nose; left/right mastoids). A palate tracer coil was used during palate-tracing sweeps (Table 3). The AG501 warmed up for approximately 15 min prior to recording. A separate bite-plane calibration recording (three embedded coils) defined the occlusal plane and enabled a head-centric coordinate frame. These bite-plane coils were not present during the speech sweeps.

**Table 3:** AG501 channel allocation used in this corpus.

Channel	Label	Anatomical target/purpose
1, 2, 3	Biteplane (L, M, R)	Occlusal plane acquisition for head correction
4, 5, 6	Ref (L mastoid, nose, R mastoid)	Head-movement correction and head-centric frame
16, 17	UL, LL	Upper and lower lip (vermilion border)
18	LC	Lip corner (left)
19, 20	UI, LI	Upper and lower incisors (jaw proxy)
21, 22, 23	TD, TB, TT	Tongue dorsum, blade, tip (midline)
24	PT	Palate tracer (palate sweep)

Channels not listed were spares.

Head motion was removed via rigid-body correction using the reference coils, implemented with Carstens utilities cs5calcpos (position reconstruction) and cs5normpos (normalisation/head correction). In the AG501 coordinate system, *x* indexes the anterior—posterior (front—back) axis, *y* the left—right axis, and *z* the inferior—superior (down—up) axis. Orientation angles (*ϕ*, *θ*) are provided per sensor.

**Fig. 2:**
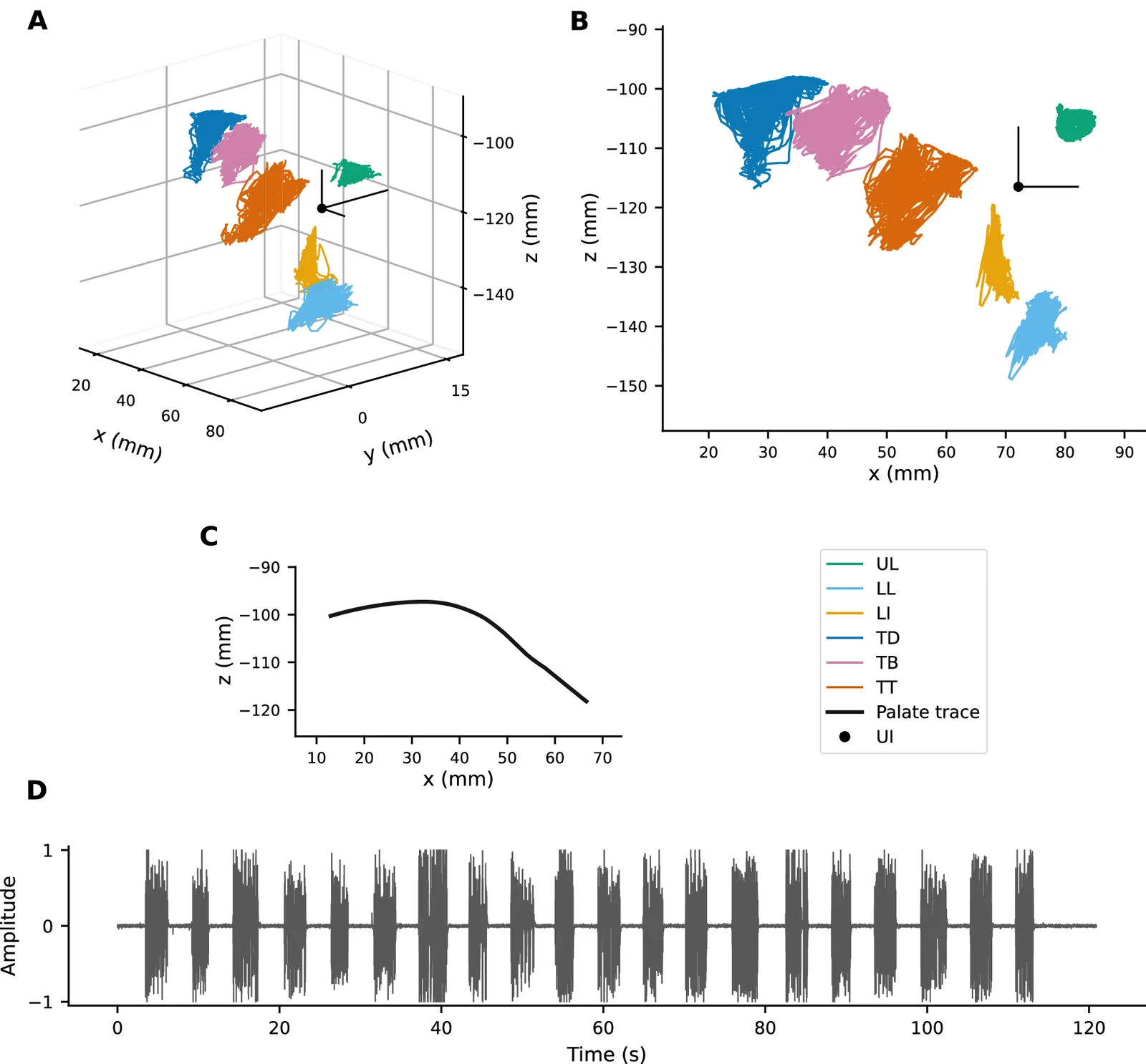
Articulatory kinematics during a representative DDK sweep. Example shown for participant sub-891. **(A)** Three-dimensional trajectories of the EMA sensors (UL, LL, LI, TD, TB, TT) in a UI-centered coordinate system. **(B)** Sagittal x-z projection of the same trajectories. **(C)** Subject-specific palate trace in the sagittal plane compiled from palate-tracing sweeps. **(D)** Corresponding audio waveform for the same sweep as in panels A and B.

To illustrate the coordinate frame, palate reference, and typical excursions, Fig. 2 shows representative 3D EMA trajectories (A), their sagittal projection (B), a participant-specific palate trace compiled from palate-tracing sweeps (C), and the corresponding audio waveform for the same sweep as in panels A and B (D).

#### Sensor placement and retention

Oral surfaces were prepared with ethanol swabs. To localise tongue-sensor targets, we used Dr. Thompson’s Sanitary Color Transfer Applicators to apply colour to the alveolar and posterior palatal regions. Participants then produced /t/ and /k/, leaving visible contact marks on the tongue. The /t/ mark defined the tongue-tip (TT) target, the /k/ mark defined the tongue-dorsum (TD) target, and the tongue-blade (TB) target was placed midway between them. A medical cyanoacrylate (Epiglu) bonded sensor bases to mucosa; a glass-ionomer (Ketac) fillet improved tongue-sensor retention. Incisor sensors were affixed to pre-cut adhesive patches; lip/reference sensors were secured with medical tape (Leukoflex). Cable runs avoided lip contact and permitted rounding (e.g., /u/), with slack taped to the cheek. If detachment or excessive RMS error occurred, the trial was repeated after re-affixation. At session end, sensors were removed and residual adhesive cleaned.

#### Audio

A t.bone EM 9600 condenser microphone was placed ~30 cm from the mouth. Signals were digitised at 48 kHz/24-bit. All audio is provided as WAV.

Two audio streams are provided and serve different purposes. First, the EEG system recorded prompt+response WAVs for all syllable-task trials (*N* = 300 per participant; habitual and maximally fast) in Audio/EEG_responses/. These files are annotated with Praat TextGrids *stored in the same folder* and sharing basenames with the WAVs. Second, where available, the EMA system saved EMA-synced per-sweep audio with 1:1 basenames to Audio/wav/ and a continuous version to Audio/wavall/. The presence of these EMA-synced per-sweep WAVs is flagged per participant in docs/metadata.csv. EMA-paired WAVs are convenient for optional post-hoc audio alignment relative to EMA sweeps; they are not required for EMA—EEG timing because sweep boundaries are encoded via TTLs in the EEG Status channel (see *Hardware synchronisation and trigger decoding*).

#### EEG

Scalp electroencephalography (EEG) was recorded with a 64-channel BioSemi ActiveTwo system (10–20 layout) at 2048 Hz. Electrode offsets were monitored throughout and maintained within approximately ±50 mV, consistent with manufacturer guidance. Participants were instructed to remain relaxed and to minimise extraneous movements (e.g., blinking, jaw clenching, swallowing) during pre-cue intervals.

EEG measures voltage fluctuations at the scalp that predominantly reflect synchronous postsynaptic activity in cortical neuronal populations. In this dataset, the millisecond-resolution EEG time series can be time-locked to stimulus cues and, via EMA-derived kinematic landmarks, to articulatory onsets.

**Fig. 3:**
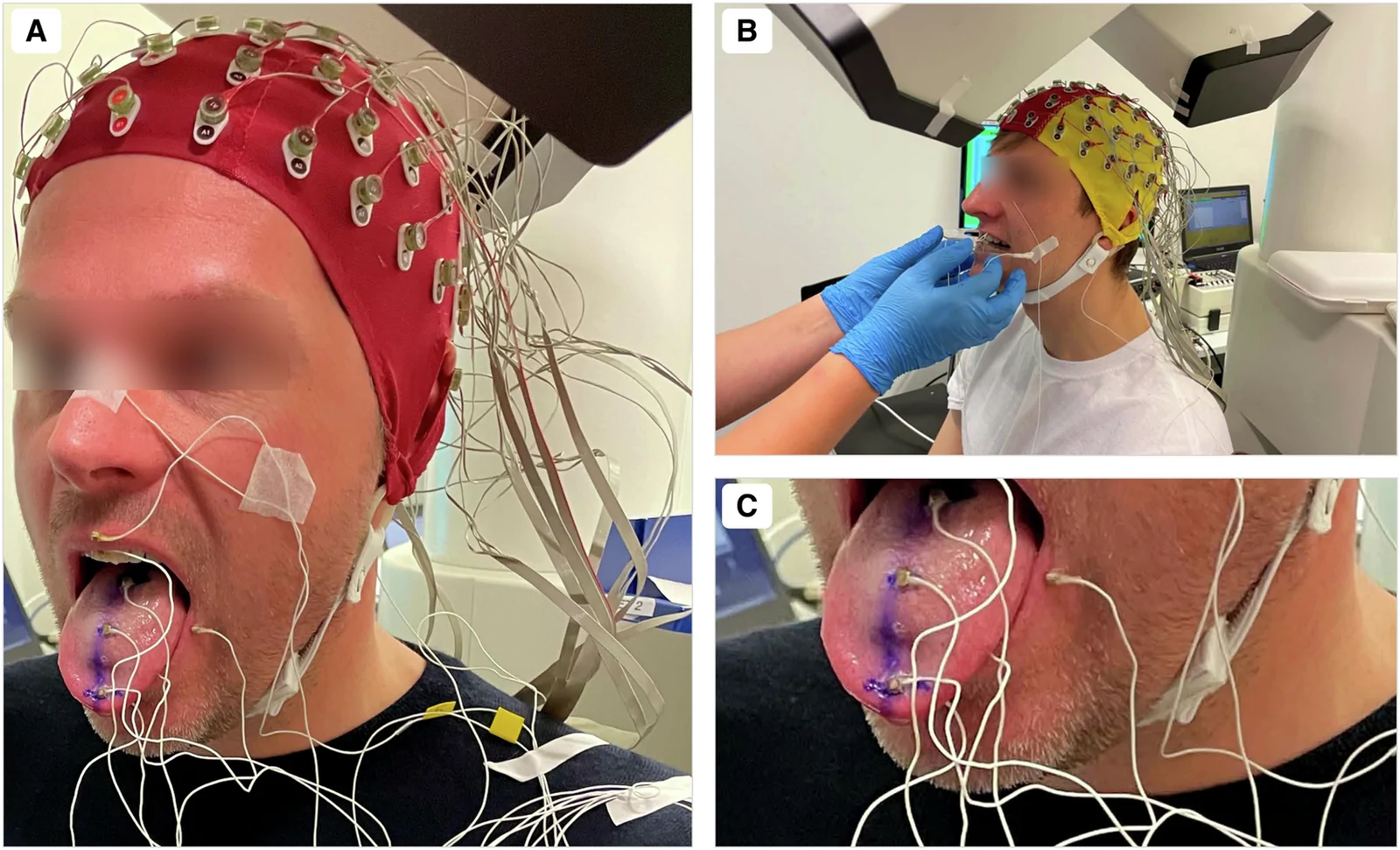
Simultaneous EMA+EEG acquisition setup. **(A)** Close-up of a participant wearing the 64-channel BioSemi EEG cap together with externally mounted EMA coils; the tongue is protruded to reveal the intraoral tongue sensors. **(B)** Wider view of a participant positioned within the Carstens AG501 transmitter during setup, illustrating the recording geometry for simultaneous EMA and EEG. **(C)** Detail view of the tongue sensors and the colour-transfer marks used to localise tongue-sensor targets. The photographs are illustrative of hardware placement and sensor configuration. Participants shown provided written consent for publication of these images, and facial regions have been blurred.

An overview photograph of the simultaneous EMA+EEG setup is shown in Fig. 3.

### Hardware synchronisation and trigger decoding

A microcontroller (Arduino Nano ESP32) monitored the AG501 T_active line and issued 1 ms TTL pulses to the BioSemi AD-box at the onset and offset of each EMA sweep. In the BioSemi *Status* (digital input) channel we used **bit 6** for sweep *start* (value 64) and **bit 7** for sweep *stop* (value 128); task codes are listed in Table 2. When a TTL coincides with a task code on the same sample, composite integers appear (e.g., 73 = 64+9). Pure task codes are recovered via *v*_core_ = *v&*63 which masks out bits 6/7 (64/128). Use rising edges on (Status & 64) and (Status & 128) for sweep start/stop, respectively. Bench oscilloscope tests measured a fixed forwarding delay of ≈2.7 ms (SD 0.4 ms) from the AG501 tap to the EEG marker; analysis scripts subtract this constant. Optional EMA-EEG audio cross-correlation (sub-sample) typically suggested 2–3 ms refinements, yielding final residual offsets of ≈0.4 ms with negligible jitter. Consistency between TTL-coded sweep boundaries and per-sweep EMA audio durations, and any residual drift, were assessed quantitatively (see *Technical Validation* for details).

**Fig. 4:**
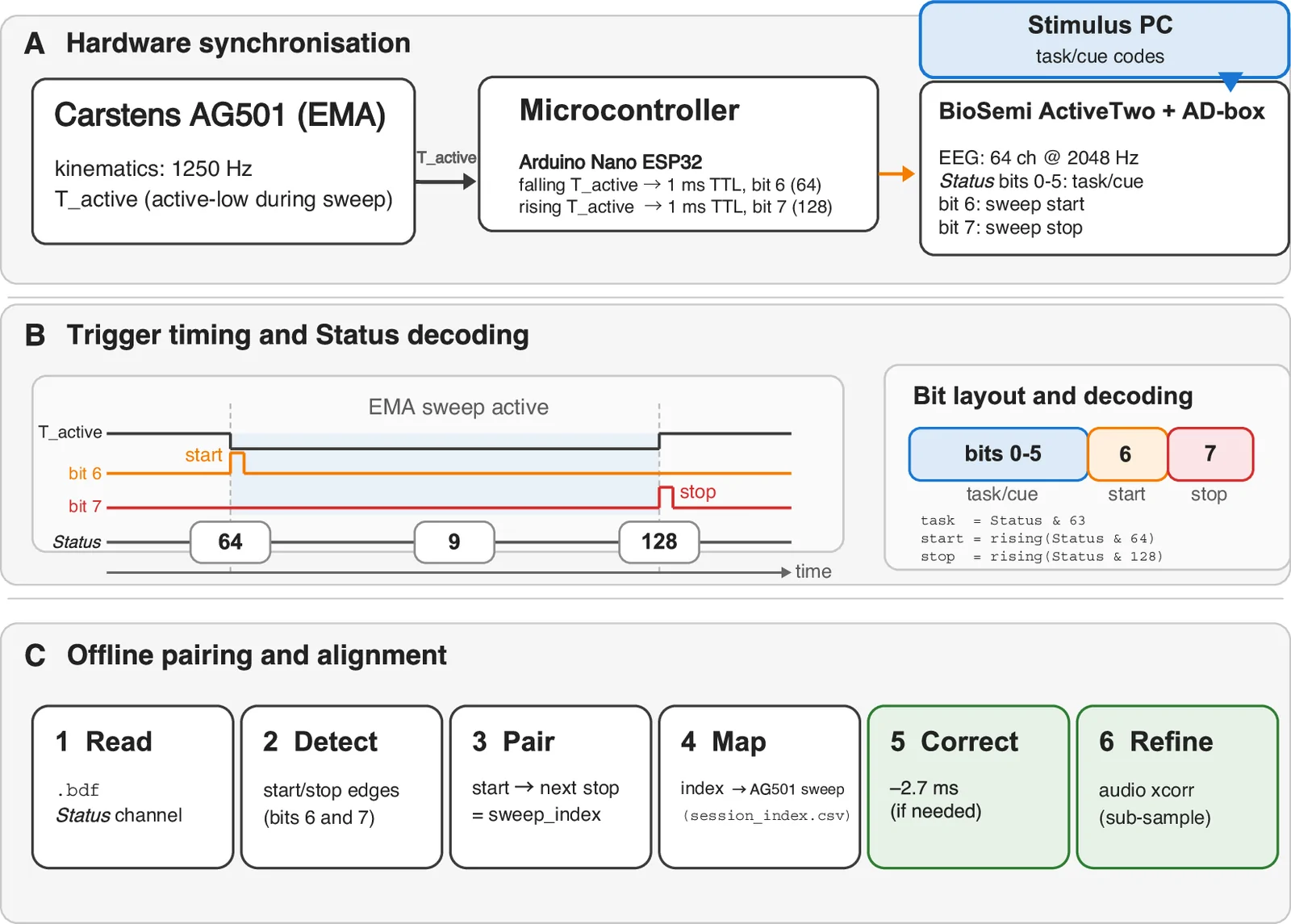
Hardware synchronisation and offline alignment of EMA and EEG. **(A)** Synchronisation hardware. The Carstens AG501 provides the sweep-state line T_active, which is active-low during each sweep. An Arduino Nano ESP32 monitors T_active and forwards sweep boundaries to the BioSemi Status channel as 1 ms TTL pulses: the falling edge of T_active is encoded on bit 6 (value 64; sweep start) and the rising edge on bit 7 (value 128; sweep stop). Task/cue codes are sent independently from the stimulus computer on bits 0–5. The microcontroller mirrors sweep boundaries only and does not control stimulus presentation or either acquisition system. **(B)** Trigger timing and Status decoding for a conceptual EMA sweep. Because TTL pulses and task/cue codes share the same Status channel, composite integers can occur when they land on the same EEG sample (e.g., 73 = 64 + 9; 137 = 128 + 9). Task/cue codes are recovered by masking out bits 6/7 (Status & 63), whereas sweep starts and stops are detected from rising edges of Status & 64 and Status & 128, respectively. **(C)** Offline pairing and alignment workflow. Read the EEG .bdf, detect start/stop TTLs, pair each start with the next stop to define sweep_index, map sweep_index to the AG501 sweep_number using session_index.csv, subtract the fixed 2.7 ms forwarding delay if needed, and optionally refine alignment by audio cross-correlation. Because each sweep is anchored by its own start/stop pair, alignment is robust to slow clock drift.

Importantly, the microcontroller does *not* start/stop either acquisition system and does not control stimulus presentation. Its sole role is to forward sweep boundary markers into the EEG recording so that EMA, EEG, and audio can be aligned *offline*. A schematic overview of the signal flow and offline alignment steps is provided in Fig. 4.

Within the EEG .bdf file, consecutive sweep start/stop pairs define a sequence of EEG-indexed sweeps (sweep_index = 0,1,2,…). For the DDK portion, each EEG-indexed sweep corresponds to one AG501 EMA sweep. To map sweep_index to the AG501 sweep_number used in the EMA file names, we use the dataset-level index in docs/session_index.csv: for each participant, we select only the DDK acquisition blocks (normal_block_* and fast_block_*), sort those rows by sweep_number, and map the nth row to sweep_index n.

### Anatomical and anthropometric measurements

A subset of 18 participants completed acoustic pharyngometry and rhinometry (Eccovision PC-3860) and a 3D head surface scan. In addition, palate tracing sweeps were acquired with an EMA palate tracer for all 29 participants, and all 29 participants completed external craniofacial anthropometry with a digital caliper.

#### Acoustic pharyngometry and rhinometry

**Fig. 5:**
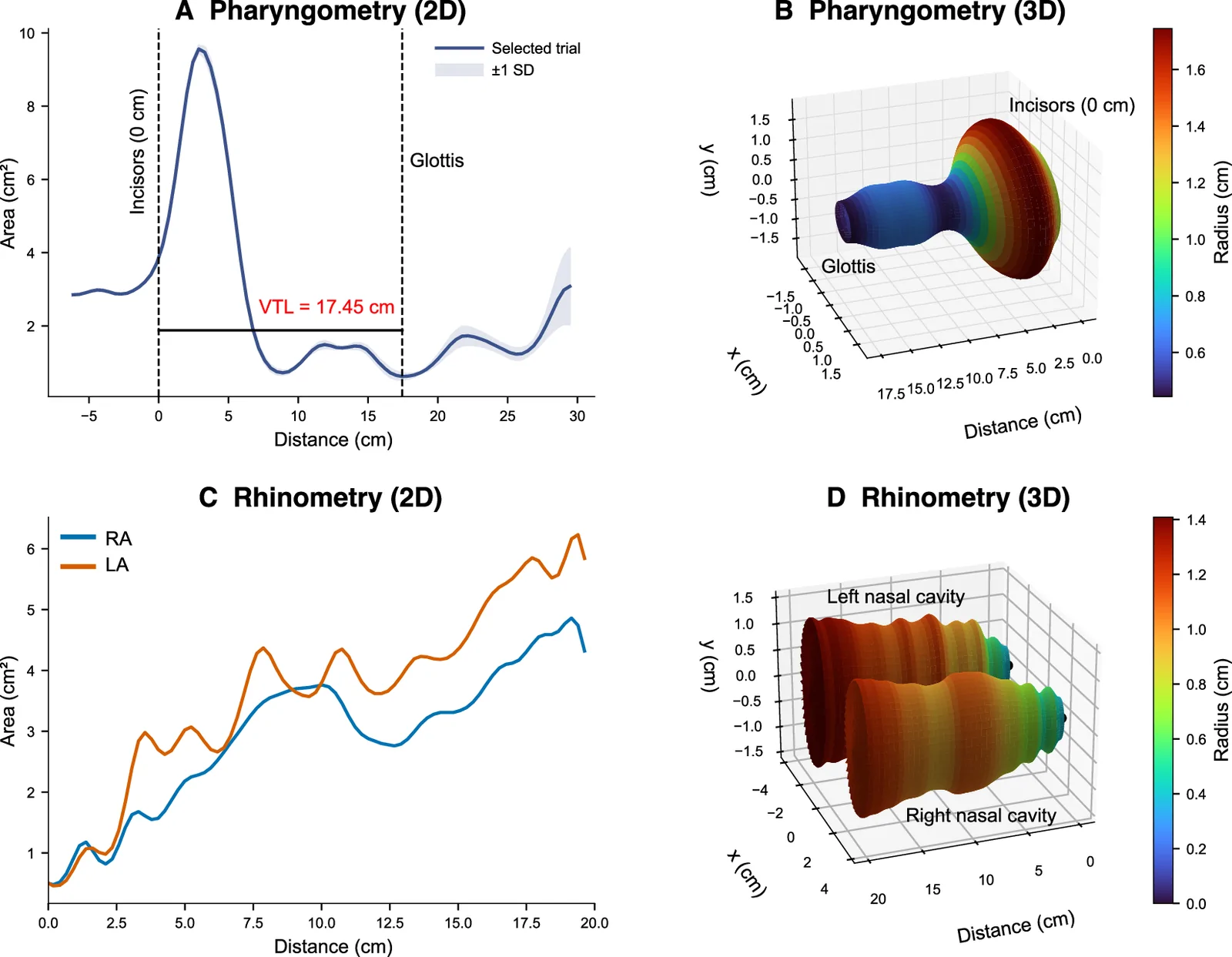
Acoustic pharyngometry and rhinometry for a representative participant (sub-989). (A) Oral-pharyngeal cross-sectional area as a function of distance from the incisors (0 cm); the shaded band shows ±1 SD across valid sweeps. Dashed lines mark the incisors and the estimated glottis. The horizontal bar indicates vocal-tract length (VTL). (B) 3D tube reconstructed from panel A between incisors and glottis; colour encodes cross-sectional radius. (C) Right and left nasal-cavity area-distance profiles from the rhinometry export (truncated at 20 cm). Curves labelled RA and LA correspond to the vendor “Right exhale” and “Left exhale” windows. (D) Corresponding 3D reconstructions of the right and left nasal-cavity profiles, laterally offset for visibility; colour encodes cross-sectional radius. Distances are in centimeters; areas in cm^2^.

Acoustic reflection methods were used to characterise upper-airway geometry: acoustic pharyngometry yields oral-pharyngeal area-distance curves, and acoustic rhinometry yields nasal area-distance curves. Measurements were acquired with participants seated upright following standard Eccovision protocols, with repeats collected until the target number of valid sweeps was obtained. For pharyngometry, each participant completed three conditions (COND1—COND3) with a target of four valid repetitions per condition; invalid trials (e.g., swallow, seal failure) were immediately re-run. Device-native waveforms were exported with the distance origin set to 0 cm at the incisors. For rhinometry, participants performed the vendor’s standard in—out protocol with a target of ≥3 valid measurements per side (right and left nasal cavities measured separately; failed attempts were repeated). Data are provided as device-native Eccovision exports in .000 format and are stored under sub-XXX/Anatomy/Pharyngometry/ and sub-XXX/Anatomy/Rhinometry/. File names follow a single, participant-ID based convention: pharyngometry is sub-XXX-phar-cond1.000, sub-XXX-phar-cond2.000, sub-XXX-phar-cond3.000; rhinometry is sub-XXX-rhino.000. In this dataset, pharyngometry exports (sub-XXX-phar-cond{1,2,3}.000) are stored in a numeric multi-block table format: each file contains four repetitions per condition in a single table (header “4 * Dist, Mean Area, Std Dev”, i.e., 12 columns = 4 × [distance, mean area, SD]). Rhinometry exports (sub-XXX-rhino.000) use the vendor AREA: table format and include the four standard Eccovision windows in one file (columns LB, RB, RA, LA; see file footer: Window #1 left in-out, Window #2 right in-out, Window #3 right exhale, Window #4 left exhale), with accompanying SD columns. The reader in code/read_eccovision_000.py supports both formats. We note that acoustic pharyngometry and rhinometry provide one-dimensional area-distance profiles and derived summary measures (e.g., lengths/volumes) rather than full 3D vocal-tract geometries. They are therefore best interpreted as speaker-specific airway calibre profiles. Figure 5 shows example oral-pharyngeal and nasal area-distance profiles and corresponding 3D reconstructions for a representative participant.

#### 3D head scans and external anthropometry

**Fig. 6:**
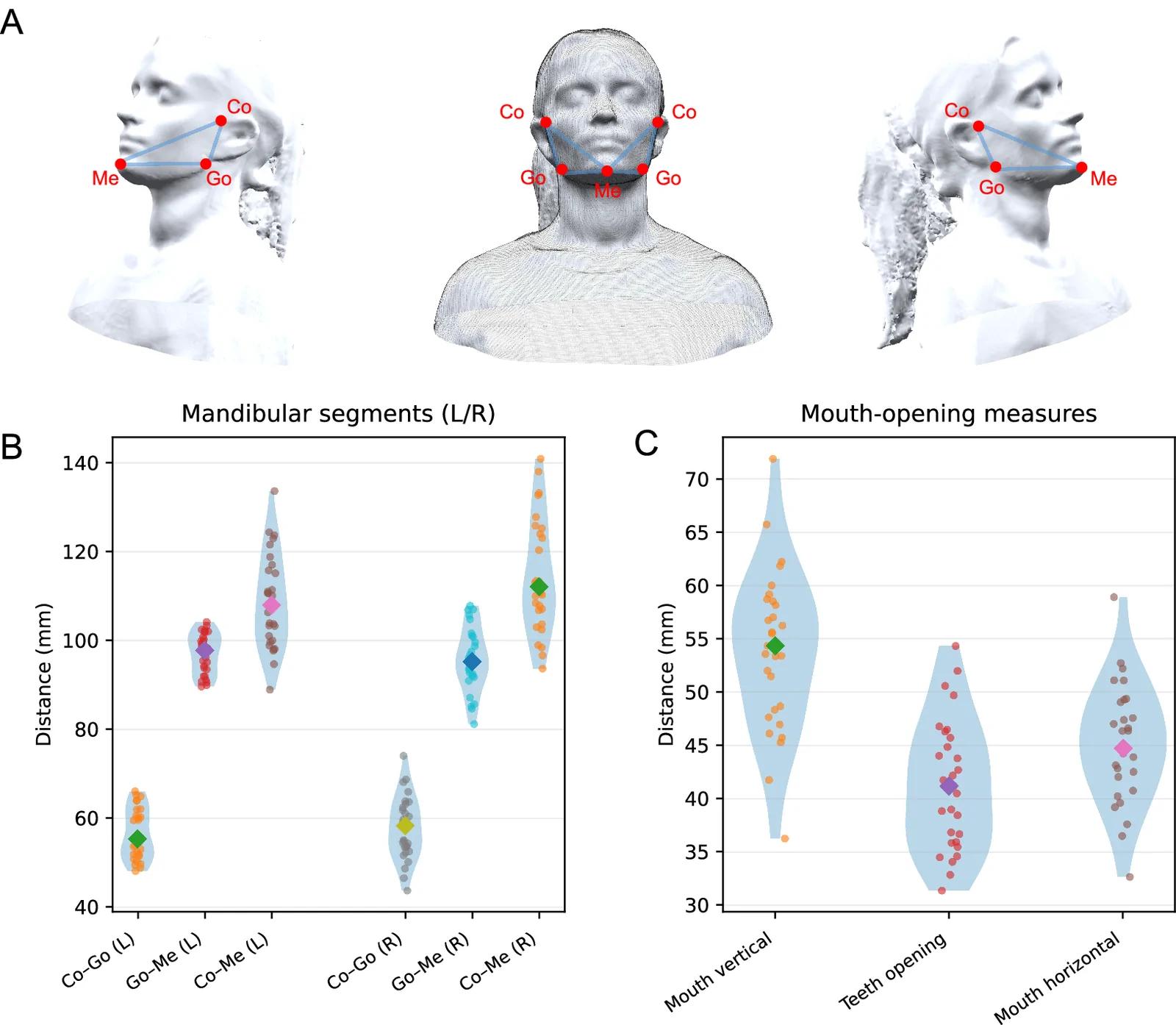
3D head surface scans and external craniofacial measurements. **(A)** Left and right sagittal views and a frontal view of an anonymised 3D head mesh (Wavefront .obj/.mtl). Mandibular landmarks are indicated: *condylion* (Co), *gonion* (Go), and *menton* (Me). For illustration, the mandibular segments Co-Go (ramus height), Go-Me (body length), and Co-Me (mandibular length) are overlaid. **(B)** Violin distributions (mm) across participants (*N* = 29) for the six caliper distances shown on the *x*-axis: “Co-Go (L)”, “Go-Me (L)”, “Co-Me (L)”, “Co-Go (R)”, “Go-Me (R)”, “Co-Me (R)”. Diamonds mark medians, and points show individual participants. **(C)** Violin distributions (mm) for other measures: maximum vertical mouth opening (“Mouth vertical”), incisor-to-incisor opening (“Teeth opening”), and maximum horizontal mouth opening. Head-size measures (head circumference, nasion–inion, ear–to–ear; reported in cm in the text but stored as mm in the CSV tables) are summarised in the text.

3D head surface scans were acquired with a Structure Sensor 3 (Occipital) mounted on an iPad using the vendor app’s static-head workflow. Meshes are provided as Wavefront .obj with an accompanying .mtl file; no facial texture is provided. OBJ vertex coordinates are in metres (SI units), consistent with typical structured-light scan exports; users should treat the mesh as a metric surface in m. External anthropometry targeted three mandibular landmarks—*condylion* (Co), *gonion* (Go), *menton* (Me)—to obtain bilateral Co—Go (ramus height), Go—Me (mandibular body), and Co—Me (mandibular length). We also recorded maximum vertical mouth opening, incisor-to-incisor ("teeth”) opening, and maximum horizontal mouth opening, together with head circumference, nasion—inion, and ear-to-ear (measured with a tape measure over the vertex). All craniofacial measurements were obtained physically on the participant using calipers and tape measures (not computed from the 3D meshes). In the released anatomy tables (docs/anatomy_measurements.csv and sub-XXX/Anatomy/sub-XXX_anatomy_measurements.csv), all length measures are stored in millimetres in value_mm (with inches in value_in). Ear-to-ear follows the curved head surface; this is consistent with the reported magnitudes. Figure 6A illustrates the mandibular landmarks used for caliper measurements; group-level distributions appear in Fig. 6B,C.

### Preprocessing and file formats

#### EMA processing

AG501 recordings were converted from vendor binaries to coil positions with cs5calcpos and normalised/head-corrected with cs5normpos. For each sweep we provide (i) head-corrected exports in EMA/pos/ and (ii) the corresponding pre-normalisation exports in EMA/rawpos/. Each sweep is distributed both as a vendor .pos file and as a whitespace-delimited ASCII .txt file. The ASCII exports contain one sample per row; depending on the Carstens export mode, time may be provided explicitly or can be reconstructed from sample index and the fixed sampling rate (1250 Hz). Per-coil amplitude diagnostics are provided as .amp files in EMA/amps/. EMA-synced per-sweep audio in Audio/wav/ (and continuous counterparts in Audio/wavall/ as *.all.wav) share the same sweep-level basename (sub-XXX-sweep-####).

For convenience, head-corrected Cartesian position coordinates in EMA/pos/ were low-pass filtered at 20 Hz using a zero-phase (forward—backward) 4th-order Butterworth filter (implemented with scipy.signal.filtfilt). We chose a 20 Hz cutoff because speech EMA trajectories are commonly smoothed with cutoffs in the 10–20 Hz range and because the dominant energy of speech articulator kinematics is typically below 15–20 Hz^17–19^; the filter therefore suppresses high-frequency measurement noise while preserving speech-relevant motion for typical kinematic analyses. Users who wish to explore higher-frequency components, or to apply alternative head-correction/filtering strategies, can start from the unfiltered native-rate 1250 Hz EMA/rawpos/ exports and the included processing logs/provenance files. These native-rate exports may be particularly useful for signal-processing-heavy applications such as articulation-to-speech synthesis^20,21^.

#### EEG formats and minimal preprocessing

Raw EEG is provided in BioSemi .bdf with event markers; derivative files in .set (EEGLAB) and .fif (MNE) are included for convenience. Minimal example preprocessing (band-pass 0.5–40 Hz; notch at 50/100/150 Hz; average reference; independent component analysis (ICA)-based ocular component removal) is illustrated in the accompanying scripts.

#### Audio

Per-sweep audio (.wav; recorded at 48 kHz/24-bit) is aligned 1:1 with EMA sweeps. Short prompt-response audio for syllable tasks resides in Audio/EEG_responses/ and is annotated with Praat TextGrids (see *Data Records*).

## Data Records

The data described here are deposited on the SWISSUbase research data repository as a version-specific release^22^; a canonical SWISSUbase record resolves to the latest version of the dataset family^23^. The repository is organised by participant (sub-XXX) and modality, with dataset-level documentation in docs/ and example analysis code in code/. EEG covers syllable (DDK) tasks at habitual and maximally fast rates; additional EMA + audio tasks broaden articulatory contexts (passage reading, sustained vowels in isolation and context). Structural/anatomical records include palate tracing (EMA) for all participants; an anatomical subset further provides oral—pharyngeal and nasal area—distance profiles (pharyngometry/rhinometry) and 3D head meshes. External craniofacial anthropometry is available for all participants in Anatomy/sub-XXX_anatomy_measurements.csv within each sub-XXX folder (Co—Go, Go—Me, Co—Me L/R; mouth openings; head-size measures; optional stature/body mass). For convenience, the deposit also includes a dataset-level summary table docs/anatomy_measurements.csv mirroring the per-participant schema. All machine-readable metadata live in docs/: metadata.csv includes demographics, screening fields, language background variables (recorded language, native language, mother/father language, dialect, and early multilingual exposure), and modality flags; event_codes.csv maps numeric task codes to labels; session_index.csv links tasks/blocks to EMA sweep numbers; and laterality_items.csv encodes hand/foot/eye preferences in long format (responses coded as 1 = left and 2 = right). Participant archives are packaged separately in the repository where the SWISSUbase interface exposes file-level selection; otherwise, the same participant folders are obtained after downloading and unpacking the full deposit.

### Task inventory and modalities

The corpus covers tightly time-locked syllable tasks (DDK; EEG available) and additional EMA + audio materials for broader articulatory contexts. Table 4 provides a compact summary of planned repetitions by task group and modality; full row-level task details are provided in Supplementary Table 1. Actual completions can vary slightly due to replanted sensors or repeats logged in the session notes. Rate labels in this manuscript are *habitual* and *maximally fast*.

**Table 4:** Task inventory and modalities.

Task group	Materials / notes	Reps	EMA	EEG	Audio
DDK, habitual	AMR voiceless, AMR voiced, and SMR sequences (nine sequence types)	9 × 20 = 180	*✓*	*✓*	*✓*
DDK, maximally fast	AMR voiceless and SMR sequences (six sequence types)	6 × 20 = 120	*✓*	*✓*	*✓*
Passage reading	*North Wind and the Sun*: one baseline read without EMA; two reads with EMA	1 + 2	mixed	—	*✓*
Sustained vowels	Eight long German vowels in isolation and in the carrier frame */l-V-gen/*	2 per vowel/context	*✓*	—	*✓*
Oromotor control	Maximal tongue protrusion and swallowing	2 each	*✓*	—	—
Palate tracing	Palatal surface sweeps with tracer sensor (PT)	2	*✓*	—	*✓*
External anthropometry	Mandibular, mouth-opening, and head-size measures	1	—	—	—
Anatomy subset (*N* = 18)	Pharyngometry, rhinometry, and 3D head scan	see text	—	—	—

Counts are planned repetitions per participant; full row-level task details are provided in Supplementary Table 1. Symbols: *✓* = available; — = not recorded. Rate strings normal/fast in filenames correspond to *habitual*/*maximally fast*. For the passage reading row, “mixed” indicates that the baseline reading is audio-only, whereas the two subsequent readings include EMA+audio. All length measures in the released anatomy tables are stored in millimetres in value_mm (with inches in value_in).

### Repository layout and naming

On SWISSUbase, directories are packaged as ZIP archives. After unpacking, files are organised by participant (sub-XXX) and modality. Each participant folder contains modality-specific subfolders for EEG/, Audio/, EMA/, and Anatomy/ where available; dataset-level documentation and metadata are stored in docs/, and example analysis scripts and environment files are stored in code/. Within each participant, EMA, audio, and EEG are partitioned by acquisition stream and aligned via sweep-level basenames and TTL codes. The full path-by-path repository inventory is provided as Supplementary Table 2.

#### Naming conventions and examples

- **Sweep-level basenames (EMA ↔ audio):** EMA files use sub-XXX-sweep-#### basenames (e.g., sub-125-sweep-0004.pos); when present, per-sweep WAVs in Audio/wav/ share the same basenames (e.g., sub-125-sweep-0004.wav).
- **EEG triggers:** TTL sweep markers are on bits 6/7 (values 64/128) of the BioSemi Status channel; task codes follow Table 2.
- **Prompt-response WAVs (DDK tasks):** <ID>-<sequence>-<rate>-<index>.wav with a matching .TextGrid sharing the same basename (e.g., sub-125-tatata-fast-41.wav and sub-125-tatata-fast-41.TextGrid) in Audio/EEG_responses/. In filenames, normal/fast correspond to *habitual*/*maximally fast*. Here, index is the stimulus-script trial counter within the corresponding rate session (habitual/maximally fast); because trial types are interleaved, indices for a given sequence are not necessarily contiguous.
- **Pharyngometry:** sub-XXX/Anatomy/Pharyngometry/sub-XXX-phar-cond{1,2,3}.000.
- **Rhinometry:** sub-XXX/Anatomy/Rhinometry/sub-XXX-rhino.000.

### Record types and file formats

#### EEG

Raw EEG is supplied in BioSemi .bdf with triggers in the Status channel (sweep start/stop TTLs on bit 6/bit 7; task codes per Table 2). Example derivatives (.set, .fif) are provided as usage exemplars.

#### Audio

Where present, per-sweep WAVs in Audio/wav/ share basenames with EMA sweeps; continuous per-sweep audio is in Audio/wavall/ as *.all.wav. Prompt-response audio for DDK tasks (*N* = 300 WAVs per participant) resides in Audio/EEG_responses/ and is annotated by matching Praat TextGrids located in the same folder.

#### EMA

Head-corrected positions are provided in EMA/pos/ as vendor .pos and ASCII .txt (1250 Hz; mm and degrees). A short columns.txt documents the ASCII column order and units. Raw (pre-correction) positions are in EMA/rawpos/; coil amplitudes for QC are in EMA/amps/. We also retain Carstens intermediate/diagnostic artefacts for provenance: *.rig, *pretrans.bin/.info/.txt, *refobj.bin/.log, *refDistances.txt, *rigidbodyana.log, *taxdistances.txt, and the vendor pattern blob sub-XXX-sweep-pattern. These are not required for typical analyses but permit re-running vendor processing and auditing head correction (Table 5).

**Table 5:** AG501 files included with each participant and their purpose.

Filename pattern	Purpose / notes
EMA/pos/*.pos, *.txt	Head-corrected positions (1250 Hz; mm; deg)
EMA/rawpos/*.pos, *.txt	Raw positions before head correction
EMA/amps/*.amp	Per-coil signal amplitudes (diagnostic)
EMA/pos/*.rig	Rigid-body solution for head-centric transform
EMA/pos/*pretrans.bin/.info/.txt	Pre-transform blob + sidecars used in normalisation
EMA/pos/*refobj.bin/.log	Reference object (bite-plane/ref-coils) + log
EMA/pos/*refDistances.txt	Inter-reference distances (stability check)
EMA/pos/*rigidbodyana.log	Rigid-body analysis log (fit residuals)
EMA/pos/*taxdistances.txt	Vendor diagnostic distances
EMA/pos/sub-XXX-sweep-pattern	Vendor pattern blob read by Carstens tools

#### Anatomy

Pharyngometry and rhinometry are provided as device-native Eccovision .000 exports named with the participant ID: Pharyngometry/sub-XXX-phar-cond1.000, sub-XXX-phar-cond2.000, sub-XXX-phar-cond3.000; and Rhinometry/sub-XXX-rhino.000. 3D head meshes are provided as .obj/.mtl (metres). External anthropometry (digital calipers; Co—Go, Go—Me, Co—Me L/R; mouth openings; head size; optional stature/body mass) is stored per participant in Anatomy/sub-XXX_anatomy_measurements.csv. A dataset-level summary docs/anatomy_measurements.csv mirrors the same schema.

#### Annotations

Praat TextGrids stored in Audio/EEG_responses/ (basenames matching the WAVs) mark visible cue onset and response onsets for AMR/SMR tasks at both rates.

#### Metadata and machine-readable maps

docs/metadata.csv provides demographics and session-level modality flags (see docs/metadata_dict.json and docs/README_metadata.md). docs/event_codes.csv maps numeric task codes to labels. docs/session_index.csv links tasks/blocks to EMA sweep numbers, enabling programmatic pairing across streams. docs/laterality_items.csv encodes hand/foot/eye preferences in long format.

## Data Overview

Across 29 speakers, this release contains ~17 h of EMA sampled at 1,250 Hz with duration-matched 48 kHz audio where available, and ~29 GB of raw 64-channel EEG sampled at 2,048 Hz for DDK tasks. Twenty-five participants have tri-modal coverage for the DDK tasks (EMA+EEG+*EMA-synced* per-sweep audio), 26 participants have *EMA-synced* per-sweep audio, and all participants have EEG-system prompt+response WAVs (300 files each). These completeness cases are intentionally retained for reuse and are explicitly flagged in docs/metadata.csv. Machine-readable indices map tasks and blocks to EMA sweep identifiers and EEG TTLs, enabling programmatic pairing. The anatomical subset (*N* = 18) contributes oral—pharyngeal and nasal area—distance profiles and 3D head meshes; all 29 participants contribute palate tracing and external craniofacial anthropometry.

## Technical Validation

### Synchronisation validation (durations, drift, mismatch)

**Fig. 7:**
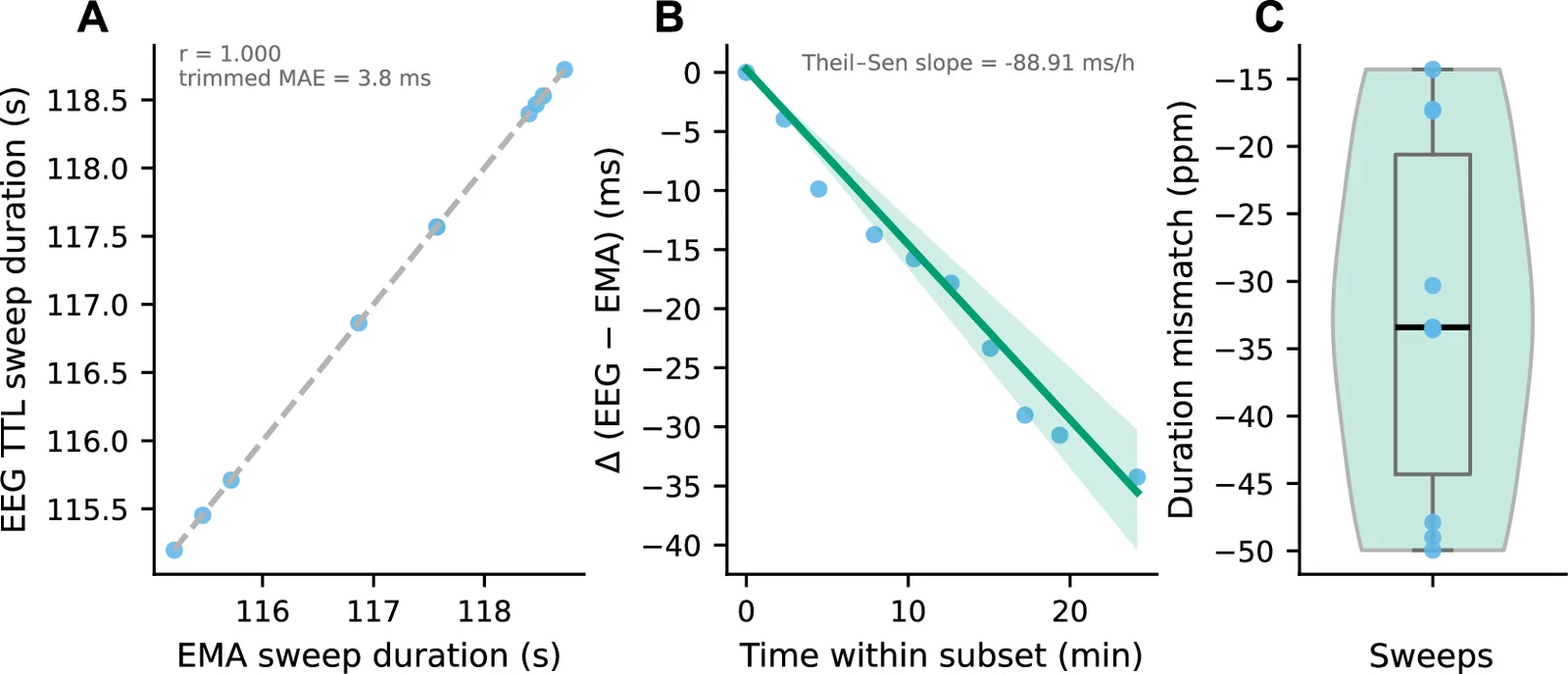
Synchronisation validation (example: sub-125). **(A)** Per-sweep durations from EEG TTL start-stop pairs versus EMA audio-derived durations (identity line; trimmed MAE = 3.8 ms). **(B)** Residual drift *Δ* = EEG-EMA within an aligned subset with Theil-Sen slope (95% CI; −88.91 ms/h). **(C)** Duration mismatch (ppm) across sweeps; median −33.4 ppm indicates the EEG clock runs ~32-33 ppm slower than EMA. Per-sweep TTL anchoring, together with fixed-delay correction and optional audio refinement, yields sub-millisecond final event alignment despite the small relative clock bias.

We validated EMA-EEG timing by comparing per-sweep durations, estimating any residual drift within aligned subsets, and quantifying relative clock-rate mismatch. In the representative session shown in Fig. 7, EEG TTL start-stop durations closely matched EMA audio-derived durations (*r* ≈ 1.00; trimmed mean absolute error = 3.8 ms); cumulative drift *Δ* = (EEG-EMA) followed a small negative Theil-Sen slope of −88.91 ms/h (95% CI shown); and duration mismatch had a median of −33.4 ppm. This indicates that the EEG clock runs ~32–33 ppm slower than the EMA reference (about 0.12 s per hour), but because each sweep is anchored by its own start/stop TTLs, and event-level alignment additionally uses fixed-delay correction and optional audio refinement, the final event-alignment error remains sub-millisecond.

To verify that the 20 Hz low-pass filter used for the distributed head-corrected EMA position traces does not materially affect derived articulatory-onset estimates, we repeated onset detection using an otherwise identical pipeline with a 30 Hz cutoff. Across a representative subset of DDK sweeps and sensors (TT/TB/TD), onset estimates were highly consistent across cutoffs (median absolute difference: 0.8 ms; 95th percentile: 2.4 ms). This comparison concerns the robustness of the derived EMA onset marker rather than the underlying EMA-EEG synchronisation, which is validated separately.

### Calibration stability and electromagnetic cross-talk

We evaluated whether the presence of the EEG apparatus had any impact on the EMA system’s calibration accuracy (and vice versa). To do this, we performed the standard EMA spatial calibration procedure under three different conditions: (i) with no EEG equipment present, (ii) with the EEG cap worn by a participant but the amplifier turned off, and (iii) with the EEG cap on and the system actively recording. We used the AG501’s built-in circular calibration routine (as described by Kirkham *et al*.^24^) for each condition. In each case, we collected multiple sensor rotations through the measurement volume (three full rotations per condition, using all 24 sensors, for a total of nine calibration runs).

After each calibration run, we computed metrics to quantify the calibration quality. In particular, for each sensor, we examined the peak-to-peak range of its recorded *z*-axis movement (*Δz*) and the standard deviation of its *z* position (*σ*_*z*_) during the known circular motion. Any increase in these values would suggest a loss of accuracy or consistency. We then used a Bayesian mixed-effects model (with sensor as a random effect) to compare the metrics across the three conditions. Our analysis found no credible differences in either *Δz* or *σ*_*z*_ when the EEG cap was introduced. In fact, the results favored the null hypothesis (Bayes factor *BF*_10_ = 0.04 for the comparison between the no-EEG and EEG-on conditions), indicating strong evidence that the head-mounted EEG electrodes did not measurably degrade the EMA’s spatial precision. In practical terms, the EMA calibration remained stable and within normal bounds (with peak-to-peak ranges and variances comparable to those observed without any EEG equipment), confirming that simultaneous use of the EEG cap does not compromise the articulatory data quality.

### EEG spectral purity during transmitter operation

To evaluate whether the EMA transmitter introduced electromagnetic interference into the EEG recordings, we collected baseline noise data with an EEG cap and electrodes submerged in a water bucket positioned beneath the EMA system, thereby approximating the electrical properties of the human head in the absence of biological signals. EEG was recorded at 2048 Hz while the EMA magnetic field was alternately inactive (transmitter off) and active (transmitter on). For each condition we computed power spectral density (PSD) estimates over 0–1024 Hz using Welch’s method (Hann windows; 50% overlap). We then compared transmitter-off and transmitter-on PSDs using a cluster-based permutation approach across contiguous frequency bins: at each frequency bin we computed a paired-samples test statistic for the on-off contrast, formed clusters of adjacent bins exceeding a sample-level threshold of *p* < 0.05, and defined cluster mass as the sum of test statistics within each cluster. Cluster-level significance was assessed against a null distribution obtained from 1000 random within-channel label permutations (sign flips). As a confirmatory analysis, we additionally evaluated effects using threshold-free cluster enhancement (TFCE). This analysis found no significant difference in EEG spectral power attributable to the EMA transmitter being active: using a prespecified cluster-level significance threshold of *α* = 0.05, no frequency cluster was significant, and all observed cluster-corrected *p* values were > 0.10. We also observed no spurious peaks indicative of aliasing of the EMA carrier frequencies (9–16 kHz) into the recorded EEG band. As an additional realism check, we recorded EEG from a live participant while the EMA was running and likewise observed no discernible differences between transmitter-off and transmitter-on quiet (pre-speech) periods after standard filtering. These results confirm that operating the AG501 transmitter does not introduce any measurable electromagnetic interference into the EEG recordings.

### EEG preprocessing checks (for quality assessment)

Processing was performed in MNE-Python^25^. We applied a minimal, commonly used pipeline (band-pass 0.5–40 Hz; 50/100/150 Hz notches; average reference) and removed ocular components using ICA (Picard^26^, with optional FastICA refinement^27^; design choices following Klug and Gramann^28^). After cleaning, prestimulus RMS and channel-wise variance fell within typical laboratory bounds, and no persistent spectral lines beyond power-line harmonics remained. These checks show that the distributed raw EEG, when processed with standard methods, attains expected quality for analyses time-locked to articulatory or cue-related events. Example pipelines are provided with the code bundle (see *Usage Notes*).

### Anatomical and anthropometric data quality

Pharyngometry and rhinometry trials were repeated at least three times per participant, and outlier trials were discarded as described above. The measurements showed good consistency: intra-participant variability (e.g., the standard deviation in total volume across repeats) was generally under 8%. This indicates that the anatomical area—distance profiles are reliable for characterising individual differences in vocal-tract morphology.

All 29 participants have complete external anthropometry. We performed unit checks and verified value ranges; group-level distributions were plausible (Fig. 6B–C). Head circumference averaged 56.2 cm ± 1.5 (range 53–60 cm); nasion—inion and ear—to—ear averaged 34.9 cm ± 1.1 (34–37 cm) and 43.1 cm ± 1.7 (40–46 cm), respectively. Maximum vertical mouth opening averaged 53.9 mm ± 7.5 (36–72 mm), incisor-to-incisor opening 41.3 mm ± 6.1 (31–54 mm), and maximum horizontal opening 45.2 mm ± 5.6 (33–59 mm); the vertical exceeded incisor-to-incisor by 12.6 mm ± 7.0 on average. For the mandibular segments, left—right averages were 57.1 mm ± 5.6 for **Co—Go**, 96.3 mm ± 5.2 for **Go—Me**, and 111.5 mm ± 10.9 for **Co—Me**. Left/right agreement was typical for manual calipers: median |*L* − *R*| differences were 3.1 mm (Co—Go), 3.4 mm (Go—Me), and 4.9 mm (Co—Me), with 95th-percentile absolute differences of 10.2, 11.5, and 16.2 mm; left/right values correlated at *r* = 0.55, 0.60, and 0.75, respectively.

## Usage Notes

Because overt articulation introduces facial myogenic artifacts into EEG, many analyses will target pre-cue or pre-movement windows. EMA-EEG alignment enables time-locking to articulatory onsets (e.g., tongue-tip lift for /t/) rather than acoustic onsets. For windows that include overt speech, ICA or regression approaches mitigate myogenic artifacts; the example pipelines illustrate both. For modality-specific analyses, users can subset participants by the completeness flags provided in docs/metadata.csv.

Example scripts in code/ demonstrate how to (i) parse TTL pulses on the EEG Status channel and pair EMA sweeps (eeg_status_bits.py; parse_status_and_pair_sweeps.py; quickstart.py), (ii) read AG501 ASCII and detect articulatory onsets from EMA velocities, optionally refining timing using audio cross-correlation (ema_io.py; detect_articulatory_onsets.py; audio_sync.py), (iii) read and plot area-distance curves from pharyngometry/rhinometry (pharyngometry_rhinometry_io.py; read_eccovision_000.py), (iv) reproduce the synchronisation and anatomy plots (make_sync_validation_figure.py; plot_caliper_violins.py; make_figures.py), and (v) run the EEG preprocessing/plotting used in the paper (01_eeg_process.ipynb/.py; 02_eeg_plot.ipynb/.py). All metadata files referenced by these scripts are under docs/.

For participants with anatomical data, the measurements can be leveraged to augment speech production analyses. The provided vocal tract area profiles (from pharyngometry) and nasal cavity measurements (from rhinometry) enable investigations into how anatomical differences might influence articulation or acoustics. For example, researchers could test whether individuals with a larger oropharyngeal volume exhibit different tongue movement patterns or require longer neural planning times than individuals with a smaller volume. Similarly, one could include the anatomical metrics as covariates in statistical models to account for inter-speaker variability in articulatory kinematics or EEG features. All the raw area-distance curves and derived metrics are included, so custom analyses relating anatomy to speech behavior can be performed.

Finally, the dataset’s structure is organized for ease of use. All files for a given trial share a common identifier, making it straightforward to programmatically load and synchronise the data. For selecting or filtering trials, use the study-level tables in docs/: session_index.csv (maps tasks/blocks to EMA sweep IDs) and metadata.csv (demographics and modality flags). Participant-level Carstens processing logs are in sub-XXX/EMA/logs/. Standard software tools can be used to read the files: for example, the tabular metadata in docs/ can be loaded with pandas, and the EMA ASCII exports can be read as whitespace-delimited tables. EEG files can be opened with MNE-Python, EEGLAB, or other suites that support BDF/SET formats. We have also provided a Docker container configuration that replicates our analysis environment, which can be useful for running the example scripts or ensuring reproducibility when reprocessing the data.

### Identifying TTL pulses in BDF and aligning EMA-EEG

BioSemi stores digital inputs in a dedicated *Status* channel. In our recordings, 1 ms pulses mark EMA sweep *start* and *stop* on **bit 6** and **bit 7**, respectively (values 64 and 128). To recover them:

1. Read the BDF (e.g., with MNE-Python’s read_raw_bdf). The trigger channel appears as Status (or STI 014, depending on library version).
2. Extract the digital stream and apply bit masks: rising edges on (Status*&*64) give sweep starts; rising edges on (Status*&*128) give sweep stops. Convert sample indices to seconds using the EEG sampling rate (2048 Hz).
3. Pair each start with the next stop to form TTL-derived sweep durations. Apply the fixed hardware offset (2.7 ms) if you wish to compare absolute times.
4. Use the per-sweep *.wav files from EMA (same basenames) or the session metadata to match TTL pairs to EMA sweeps; verify by checking that TTL-derived durations equal EMA audio durations (Fig. 7A).
5. For optional refinement, cross-correlate the EMA microphone channel with the EEG auxiliary audio and apply the sub-sample correction (typically a few ms), then proceed to epoching EEG around articulatory landmarks.

Minimal example code for steps (1)—(5) is provided under code/ (notably eeg_status_bits.py and quickstart.py), together with utilities to compute ppm mismatch and Theil-Sen drift and to reproduce Fig. 7.

Because the TTL bits share the Status channel with task codes, TTLs may coincide with a task code on the same sample (e.g., 73 = 64+9; 137 = 128+9). To recover the task code only, mask out the TTL bits with ${v}_{{\rm{core}}}=v\,\& \,63$, leaving ${v}_{{\rm{core}}}\in \{0,9,11-19,31-36\}$ (0 marks a pure TTL sample). Sweep start/stop edges are robustly detected with bit masks on the raw stream, i.e. rising edges of Status *&* 64 (start) and Status *&* 128 (stop).

Occasional TTL start—stop pairs can correspond to setup, test, or aborted sweeps rather than to DDK acquisition blocks; these may appear as short durations (for example, under 10 s) and should not be blindly mapped to DDK EMA sweeps. If the number of detected TTL pairs differs from the number of DDK rows in docs/session_index.csv, inspect the main sweep table written by parse_status_and_pair_sweeps.py, particularly the duration_s and n_code_changes fields, together with the optional decoded-events table to identify which TTL pairs correspond to DDK acquisition blocks. The parser issues a warning and maps the first n detected TTL pairs to the first n DDK rows, where n is the smaller count. Users should therefore verify the mapping before analysis.

#### Working with anatomy files

Pharyngometry and rhinometry are distributed in vendor .000 format. Utilities in code/ read these files and expose area—distance curves; distances are referenced to the incisors (0 cm). To reconstruct 3D tubes, interpret the centreline coordinate as distance_cm and convert cross-sectional area to an equivalent radius via $r=\sqrt{A/\pi }$ for visualisation. For .obj/.mtl head meshes, we recommend a simple landmarking workflow in MeshLab or Blender and exporting landmark coordinates for analysis (units are metres). External anthropometry is provided per participant in Anatomy/ and summarised at dataset level in docs/anatomy_measurements.csv; see the data dictionary for column names and units.

#### Illustrative analysis pipeline (tutorial)

**Fig. 8:**
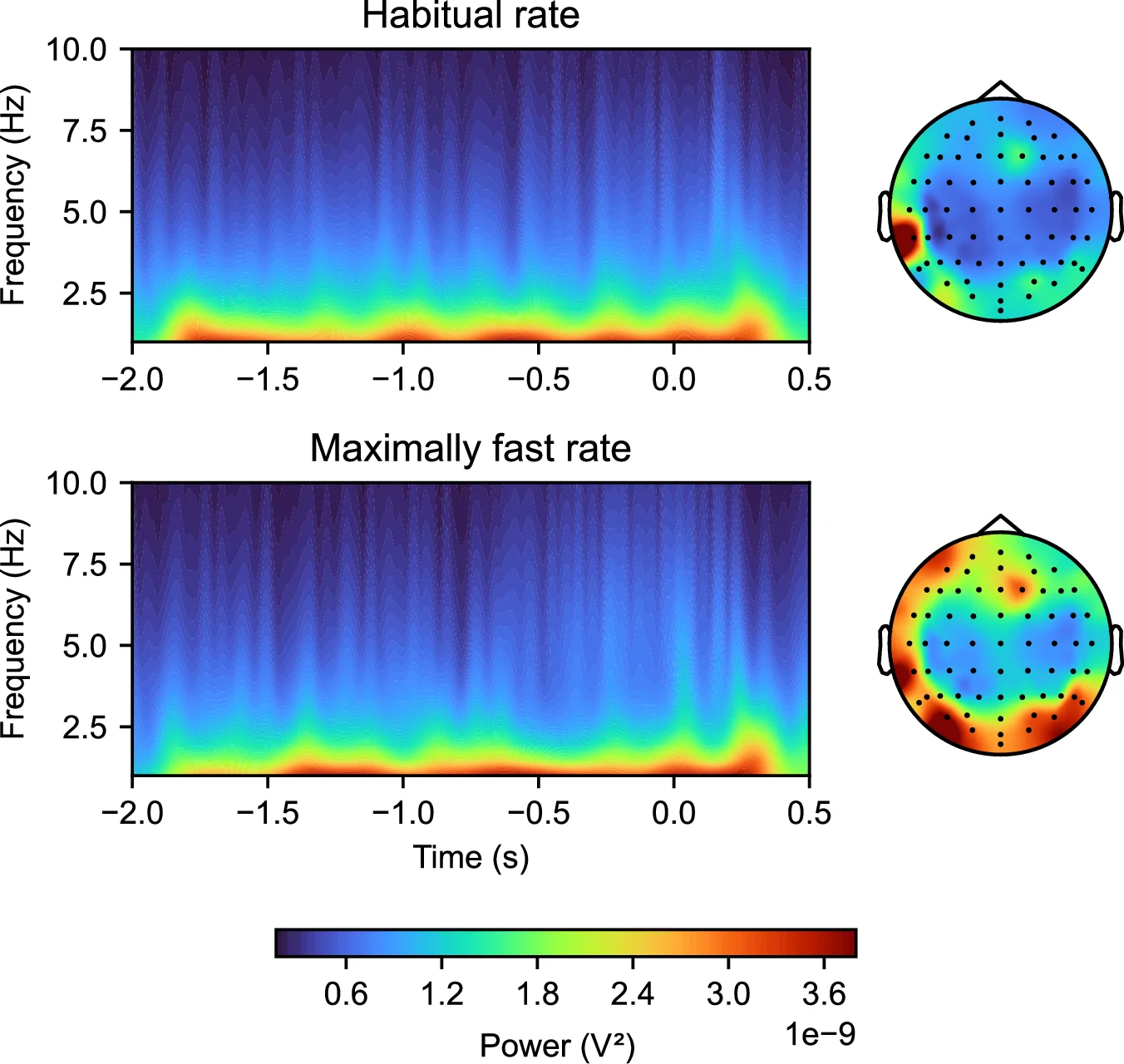
Illustrative time-frequency tutorial. Average 1–10-Hz time-frequency representations at Fz (left panels; −2.0 to 0.5 s) and scalp topographies averaged over 1–10 Hz and −1.5 to 0 s (right panels) for *habitual* (upper row) and *maximally fast* (lower row) DDK conditions. Time zero denotes the go signal. This figure is didactic only and not a claim about the dataset.

As a worked example, we provide a simple time-frequency overview for planning intervals using Morlet wavelets (1-10 Hz; 1 cycle) at a representative electrode (Fz), with scalp topographies averaged over the pre-cue window (Fig. 8). This example is solely didactic and is not intended as a result; it demonstrates typical steps (epoching to go-cues or articulatory onsets, band-pass, ICA-based ocular removal, and time-frequency estimation) that users can adapt. See 01_eeg_process.py and 02_eeg_plot.py.

## Supplementary Information


Supplementary Data 1
Supplementary Data 2


## Data Availability

All data underlying this Data Descriptor are publicly available on SWISSUbase under a CC-BY 4.0 licence. The dataset family has a canonical DOI that always resolves to the latest version (https://doi.org/10.48656/6y1s-px92); this specific release is archived with a version-specific DOI (https://doi.org/10.48656/vc7s-pt02). A human-readable project landing page is also available at https://www.swissubase.ch/en/catalogue/studies/21134/21047/overview. The deposit includes per-participant, modality-organised folders, a stable event_codes.csv, and machine-readable metadata. Brief access instructions and folder/file overviews are provided in Data Records.

## Code availability

We provide a minimal, tested Python bundle under code/ that (i) parses BioSemi Status bits and pairs EMA sweeps, (ii) aligns EMA—EEG—audio and detects articulatory onsets from EMA velocities, (iii) reads vendor pharyngometry/rhinometry files, and (iv) reproduces the validation and anatomy figures, plus an illustrative EEG preprocessing pipeline.

**Contents (key files in code/**):

• eeg_status_bits.py & parse_status_and_pair_sweeps.py — extract TTL sweep start/stop (bits 6/7) and pair them to EMA sweeps from the BioSemi Status channel.• quickstart.py — end-to-end loading: match TTL pairs to EMA sweep IDs using docs/session_index.csv; minimal alignment checks.• ema_io.py & detect_articulatory_onsets.py — read AG501 .pos/ASCII position files; compute velocities; detect articulatory onsets.• audio_sync.py — optional cross-correlation between EMA and EEG audio for sub-sample refinement.• pharyngometry_rhinometry_io.py & read_eccovision_000.py — read Eccovision .000 (pharyngometry/rhinometry) and expose area-distance curves.• make_sync_validation_figure.py — reproduce the synchronisation validation figure (per participant).• plot_caliper_violins.py — plot violin distributions for caliper and mouth-opening measures.• make_figures.py — figure panels (EMA kinematics; anatomy reconstructions).• 01_eeg_process.ipynb/.py & 02_eeg_plot.ipynb/.py — EEG preprocessing and plotting used in the paper.• validate_metadata.py — schema/consistency checks for docs/metadata.csv, docs/session_index.csv, and related dictionaries.• README_CODE.md, requirements.txt, Dockerfile — quickstart and reproducible environment files.

Environment files (requirements.txt; Dockerfile) are included to facilitate reproducibility. No external repositories are required.
